# Supplementary material for: The Gut Bacteria Dysbiosis Contributes to Chronic Graft-Versus-Host Disease Associated With a Treg/Th1 Ratio Imbalance
Source: Front Microbiol. 2022 Sep 8;13:813576. doi: 10.3389/fmicb.2022.813576 (PMC9493085; doi:10.3389/fmicb.2022.813576)
Supplement: Supplementary file 1 [file Data_Sheet_1.zip › P101SC18090073-01-B1-3-4_result/04.BetaDiversity/MetaStat/species/boxplot/figures/g__unidentified_Lachnospiraceae_s__Lachnospiraceae_bacterium_COE1.pdf]

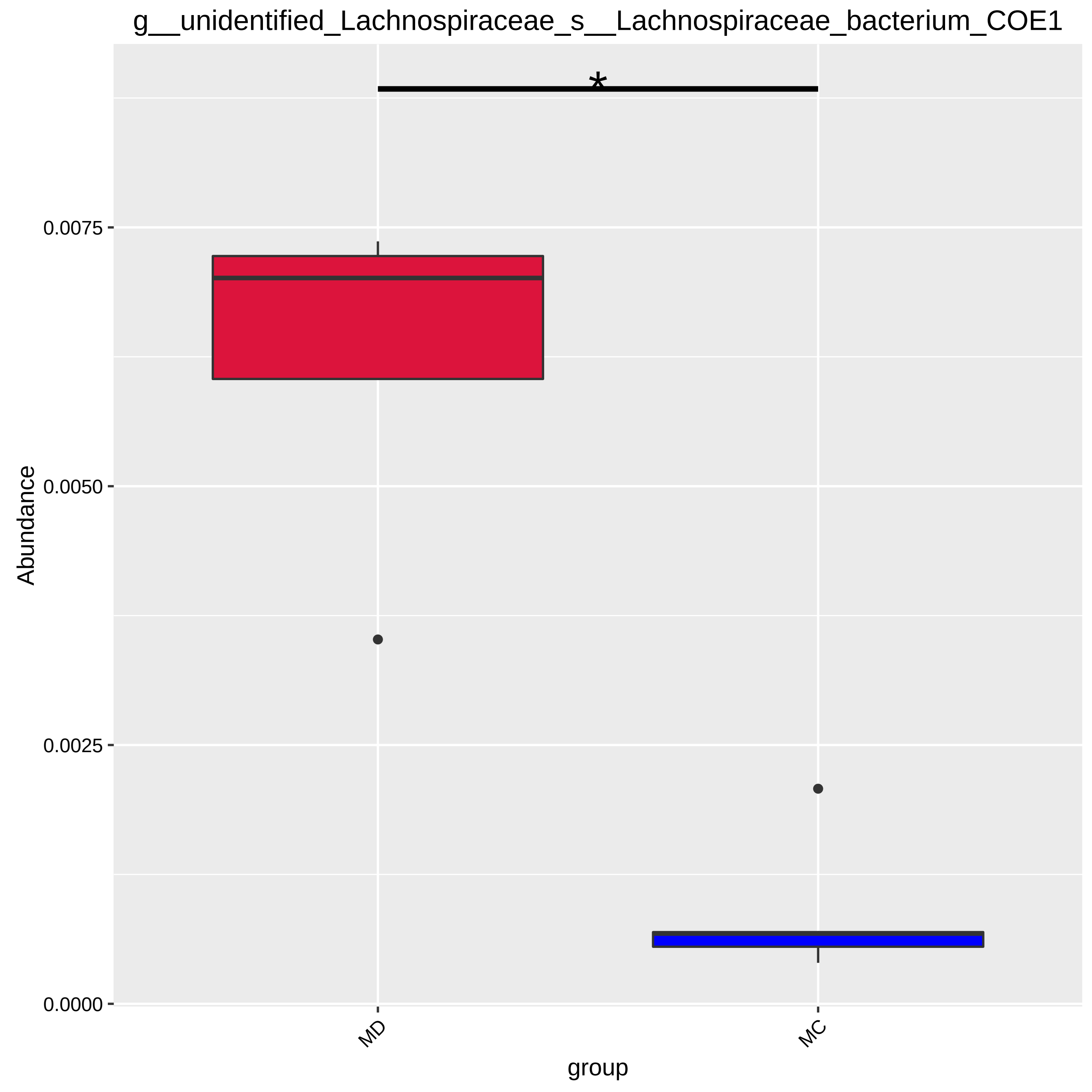

g__unidentified_Lachnospiraceae_s__Lachnospiraceae_bacterium_COE1
*
0.0075
0.0050
0.0025
0.0000
Abundance
MD
MC
group
